# ^13^C-NMR Data of Diterpenes Isolated from *Aristolochia* Species

**DOI:** 10.3390/molecules14031245

**Published:** 2009-03-23

**Authors:** Alison Geraldo Pacheco, Patrícia Machado de Oliveira, Dorila Piló-Veloso, Antônio Flávio de Carvalho Alcântara

**Affiliations:** Departamento de Química, ICEx, Universidade Federal de Minas Gerais, Av. Presidente Antônio Carlos, 6627, Pampulha, Belo Horizonte, MG, Brazil

**Keywords:** *Aristolochia*, Aristolochiaceae, Clerodanes, Furanoditerpenes, Kauranes, Labdanes, ^13^C-NMR data.

## Abstract

The genus *Aristolochia*, an important source of physiologically active compounds that belong to different chemical classes, is the subject of research in numerous pharmacological and chemical studies. This genus contains a large number of terpenoid compounds, particularly diterpenes. This work presents a compilation of the ^13^C-NMR data of 57 diterpenoids described between 1981 and 2007 which were isolated from *Aristolochia* species. The compounds are arranged skeletonwise in each section, according to their structures, i.e., clerodane, labdane, and kaurane derivatives. A brief discussion on the ^13^C chemical shifts of these diterpenes is also included.

## Introduction

The genus *Aristolochia* (Aristolochiaceae) consists of about 500 species mostly distributed along tropical, subtropical, and Mediterranean regions of the world [1,2,3]. The *Aristolochia* species are cultivated as ornamentals [4] and popularly used as sources of abortifacient, emmenagogue [5,6], sedative [7], analgesic, anticancer, anti-inflammatory, antifeedant [8], muscle relaxant [9], antihistaminic, and antiallergic [10] drugs, for intestinal worms, in the treatment of cholera, stomach ache, abdominal pain, rheumatism [11], malaria [12], wounds and skin diseases [13], and also useful in treatment of different types of poisonous bites and stings [14,15]. Several other biological properties have been described [16]. On the other hand, consumption of many plants of the genus can lead to progressive nephrophathy and urothelial cancer in humans [17,18]. As a consequence, the distribution of herbal medicines containing *Aristolochia* extracts are prohibited in many countries due to their nephrotoxic, carcinogenic, and mutagenic properties [1]. 

**Figure 1:**
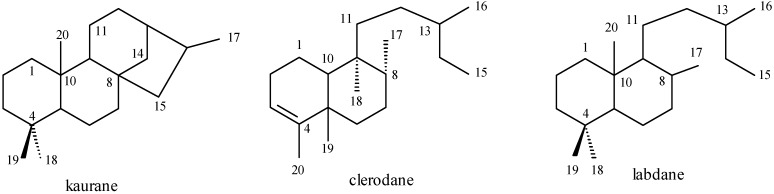
Diterpene classes present in *Aristolochia* species.

Aristolochic acids have been frequently found in *Aristolochia* species [19]. These compounds show toxic effects at the renal level and carcinogenic properties [20,21]. Phytochemical investigations of these species revealed both the presence of aporphinic, tetrahydroprotoberberinic, benzyltetra-hydroisoquinolinic, and bisbenzyltetrahydroisoquinolinic alkaloids [22] and other nitrogenated derivatives (phenantrenoids, aristolactams, and porphyrins) [23,24,25]. Quinones, coumarins, flavanoids, lignoids (phenylpropanoids, neolignans, and lignans), and fatty acids are frequently isolated from plants of the genus [26]. However, the most prominent compounds in *Aristolochia* are terpenoids, constituents of the essential oils isolated from the plant species. The majority of the identified terpenoids are kaurane, clerodane, and labdane diterpene derivatives (Figure 1). 

**Figure 2:**
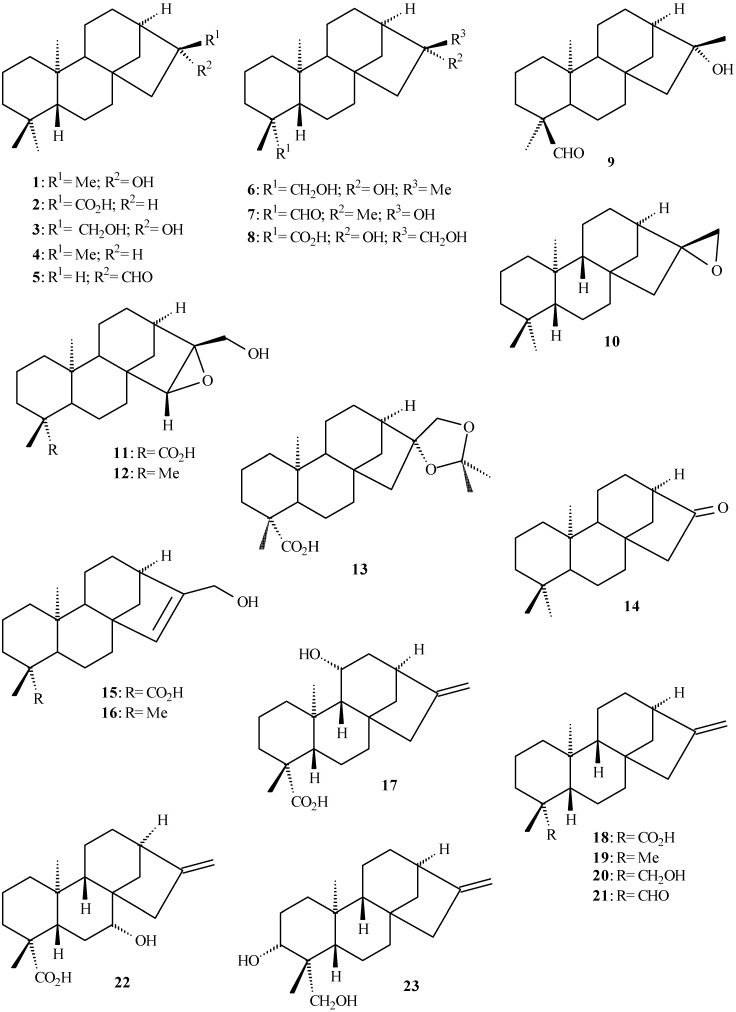
Kaurane diterpenoids isolated from *Aristolochia* species.

**Figure 3:**
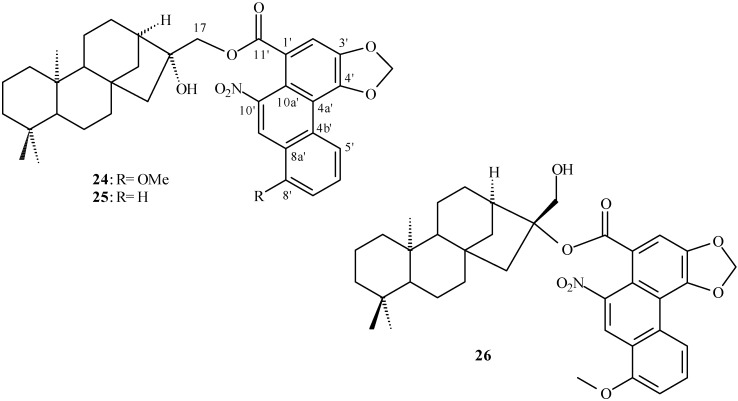
Substituted kaurane diterpenoids isolated from *Aristolochia* species.

**Figure 4:**
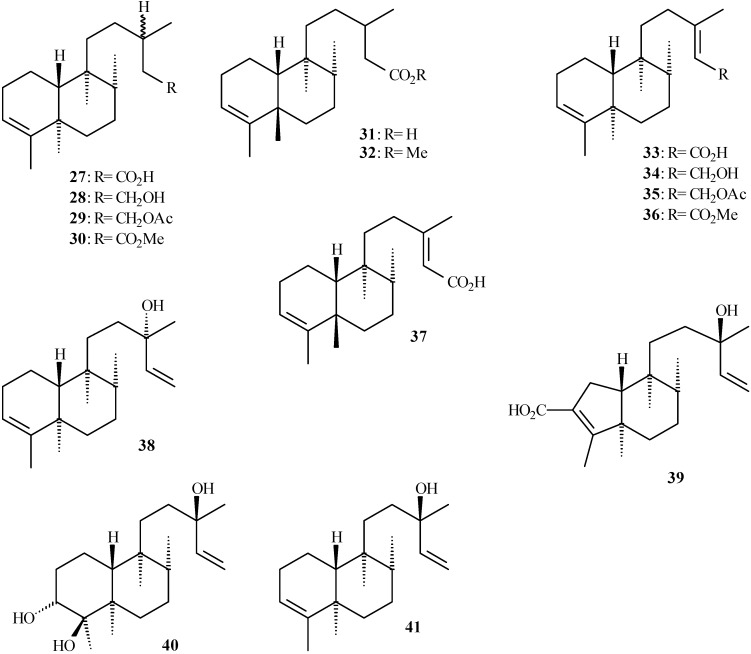
Clerodane diterpenoids isolated from *Aristolochia* species.

**Figure 5:**
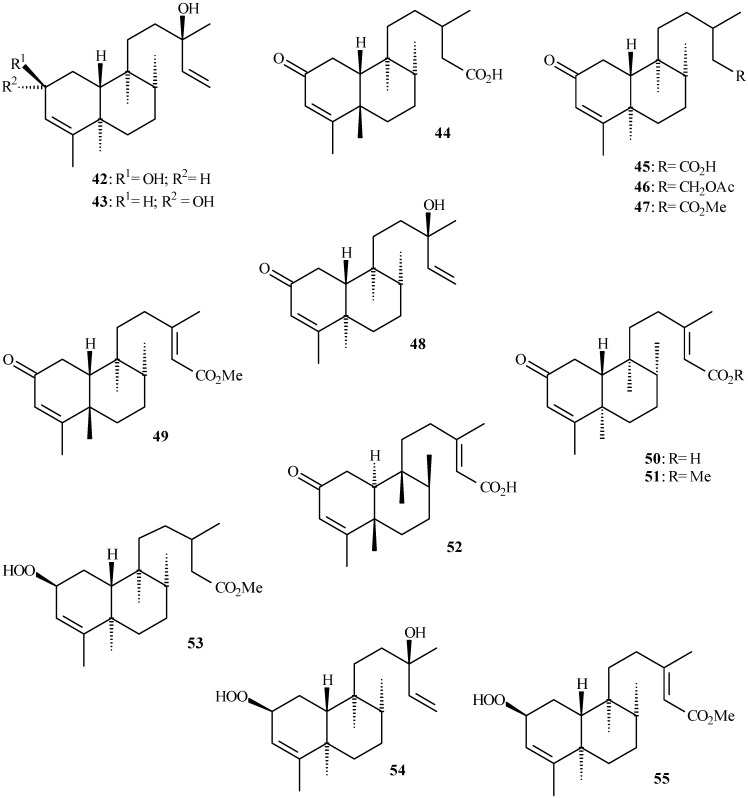
Clerodane diterpenoids isolated from *Aristolochia* species, showing oxygened C-2.

**Figure 6:**
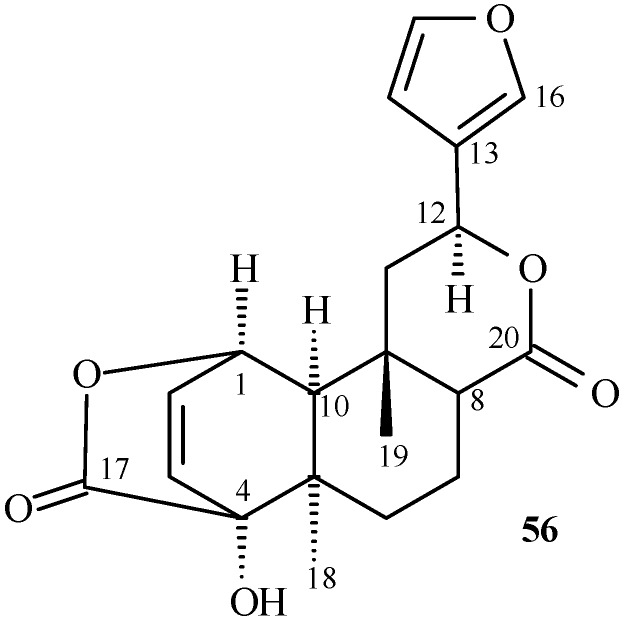
Furanoditerpene isolated from *Aristolochia* species.

**Figure 7:**
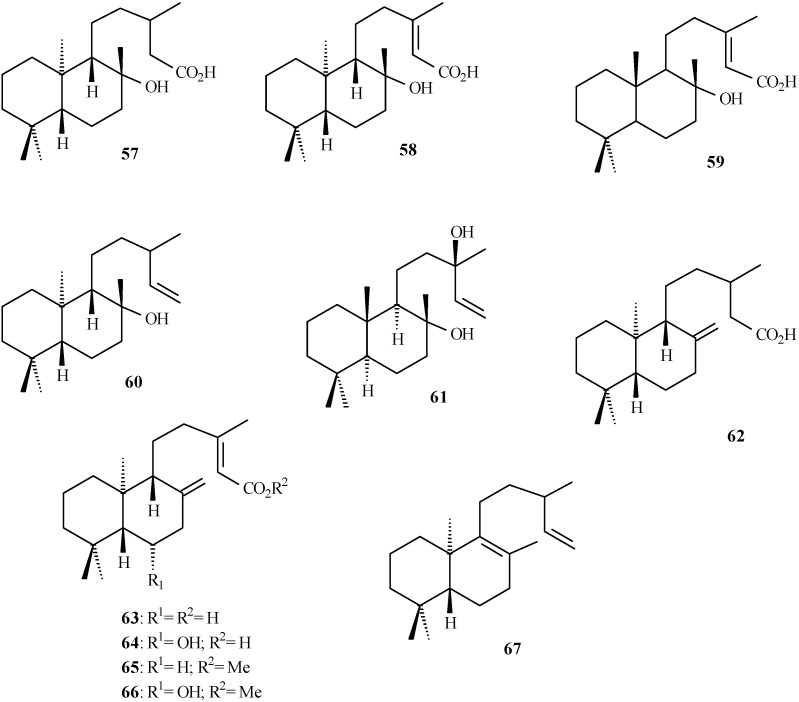
Labdane diterpenoids isolated from *Aristolochia* species.

The review “Terpenoids of *Aristolochia* and their biological activities”, which covered the literature up to 2003, lists 52 diterpenoids isolated from the genus and their pharmacological properties [16]. In the present review a new comprehensive coverage of diterpenes isolated from *Aristolochia* species up to this moment (Table 1, Table 2 and Table 3) is described, broadly covering 26 kauranes (Figure 2 and Figure 3), 29 clerodanes (Figure 4 and Figure 5), one furanoditerpene derivative (Figure 6), and 9 labdanes (Figure 7). Moreover, the ^13^C-NMR data of these compounds are also compiled (Table 4 and Table 5). For some structures no ^13^C-NMR data was found in the investigated literature and there is disagreement concerning the^ 13^C-NMR data of (–)-11-hydroxykaur-16-en-19-oic acid. Sometimes different structures were given the same names. 

## Kaurane derivates isolated from *Aristolochia* species

Kaurane diterpenoids show several biological properties such as antioxidative, antityrosinase [27], abortifacient, and anti-inflammatory activities, they are used against snake bite poisoning [28], and present cytotoxicity against tumor cells of human prostate, colon, and breast cancer [29]. Table 1 lists the kaurane derivates isolated from *Aristolochia* species (**1** to **26** in Figure 2 and Figure 3) and their respective plant sources. Acetonide **13** and kaurane derivative **14** were isolated from *A. rodriguesii* and *A. triangularis*, respectively. Both compounds were also prepared from **3 **[30]. 

**Table 1:** Kaurane diterpenoids isolated from *Aristolochia* species.

Clerodane	Species
*ent*-Kauran-16*β*-ol [(–)-kauranol] (**1**)	*A. rodriguesii* [28]
*ent*-16*β*(*H*)-Kauran-17-oic acid (**2**)	*A. elegans* [7]; *A. triangularis* [13]
*ent*-Kauran-16*β*,17*-*diol (**3**)	*A. elegans* [7]; *A. pubescens* [31]; *A. triangularis* [13]
*ent*-16*β*(*H*)-Kaurane (**4**)	*A. elegans* [7]; *A. triangularis* [13]
*ent*-16*α*(*H*)-Kauran-17-al (**5**)	*A. elegans* [7]
*ent*-Kauran-16*β*,19-diol [*ent*-16*β*,19-dihydroxykaurane] (**6**)	*A. rodriguesii* [28]
*ent*-16*α*-Hydroxy-kauran-19-al [16a-hydroxy-(–)-kauran-19-al] (**7**)	*A. rodriguesii* [28]; *A. triangularis* [32]
*ent*-16*β*,17-Dihydroxy-(–)-kauran-19-oic acid (**8**)	*A. rodriguesii* [28]
*ent*-16*β*-Hydroxy-kauran-18-al [(–)-kauran-16*α*-hydroxy-18-al] (**9**)	*A. triangularis* [26]
*ent*-16*β*,17-Epoxykaurane (**10**)	*A. elegans* [7]; *A. triangularis* [32]
*ent*-15*β*,16*β*-Epoxy-17-hydroxy-kauran-19-oic acid (**11**)	*A. rodriguesii* [28]
*ent*-15*β*,16*β*-Epoxykauran-17-ol (**12**)	*A. triangularis* [13]
*ent*-16*β*,17-Isopropylidenedioxy-(–)-19-oic acid (**13**)	*A. rodriguesii* [28]
17-*nor*-(–)-Kauran-16-one (**14**)	*A. triangularis* [13]
*ent*-17-Hydroxy-kaur-15-en-19-oic acid (**15**)	*A. rodriguesii* [28]
*ent*-Kaur-15-en-17-ol (**16**)	*A. elegans* [7]; *A. pubescens* [31]; *A. triangularis* [13,32]
*ent*-11*β*-Hydroxy-kaur-16-en-19-oic acid [(–)-11-hydroxy-kaur-16-en-19-oic acid] (**17**)	*A. anguicida* [33]
*ent*-Kaur-16-en-19-oic acid [kaurenic acid] (**18**)	*A. anguicida* [33]; *A. rodriguesii* [28]; *A. triangularis* [13]
(–)-*ent*-Kaur-16-ene (**19**)	*A. triangularis* [13,32]
(–)-*ent*-Kaur-16-en-19-ol (**20**)	*A. triangularis* [13,32]
(–)-*ent*-Kaur-16-en-19-al (**21**)	*A. triangularis* [13,32]
*ent*-7*β*-Hydroxy-kaur-16-en-19-oic acid (**22**)	*A. anguicida* [34]
*ent*-Kaur-16-en-3*β*,19-diol [*ent*-3*β*,18-dihydroxykaur-16-ene] (**23**)	*A. rodriguesii* [28]
*ent*-16*β*-Hydroxy-17-kauranyl aristolachate I [aristoloin I] (**24**)	*A. elegans* [4]
*ent*-16*β*-Hydroxy-17-kauranyl aristolachate II [aristoloin II] (**25**)	*A. pubescens* [31]
*ent*-17-Hydroxy-16*β*-kauranyl aristolachate I [aristolin] (**26**)	*A. elegans* [4]

Usual names are given in brackets

## Clerodane derivatives isolated from *Aristolochia* species

Clerodane diterpenoids show a broad spectrum of biological properties [35,36] including insecticidal activity [37]. Table 2 shows the clerodane diterpenoids isolated from the genus *Aristolochia* (**27** to **55** in Figure 4 and Figure 5) and their respective plant sources. Structure **52** has been also named as 2-oxokolavenic acid (**50**). The corresponding acid of **49** has been described by Wu *et al.* [16].

**Table 2:** Clerodane diterpenoids isolated from *Aristolochia* species.

Clerodane	Species
(5*R*,8*R*,9*S*,10*R*)-*ent*-3-Cleroden-15-oic acid [13,14-dihydrokolavenic acid; populifolic acid] (**27**)	*A. brasilienses* [38]; *A. cymbifera* [39]; *A. galeata* [40]
(5*R*,8*R*,9*S*,10*R*)-*ent*-3-Cleroden-15-ol [dihydrokolavenol] (**28**)	*A. galeata* [40]
(5*R*,8*R*,9*S*,10*R*)-*ent*-15-Ethanoyl-3-clerodene [dihydrokolavenol acetate] (**29**)	*A. galeata* [40]
Methyl (5*R*,8*R*,9*S*,10*R*)-*ent*-3-cleroden-15-oate [methyl populifoloate]) (**30**)	*A. esperanzae* [38]; *A. galeata* [40]
(5*S*,8*R*,9*S*,10*R*)-*ent*-3-Cleroden-15-oic acid [*epi*-populifolic acid] (**31**)	*A. cymbifera* [39]
Methyl (5*S*,8*R*,9*S*,10*R*)-*ent*-3-cleroden-15-oate (**32**)	*A. cymbifera* [39]
(5*R*,8*R*,9*S*,10*R*)-*ent*-Clerod-3,13-dien-15-oic acid [Δ^13,14^-kolavenic acid] (**33**)	*A. brasilienses* [38]; *A. galeata* [40]
(5*R*,8*R*,9*S*,10*R*)-*ent*-Clerod-3,13-dien-15-ol [Δ^13,14^-kolavenol] (**34**)	*A. galeata* [40]
(5*R*,8*R*,9*S*,10*R*)-*ent*-15-Ethanoyl-clerod-3,13-diene [acetyl kolavenoate] (**35**)	*A. galeata* [40]
Methyl (5*R*,8*R*,9*S*,10*R*)-*ent*-clerod-3,13-dien-15-oate [methyl kolavenoate] (**36**)	*A. esperanzae* [38]; *A. galeata* [40]
(5*S*,8*R*,9*S*,10*R*)-*ent*-Clerod-3,13-dien-15-oic acid (**37**)	*A. brasilienses* [38]
(5*R*,8*R*,9*S*,10*R*)-*ent*-Clerod-3,14-dien-13*β*-ol [(+)-kolavelool] (**38**)	*A. galeata* [40]
(5*R*,8*R*,9*S*,10*R*)-(4→2)-*abeo*-Clerod-13*β*-hydroxy-2,14-dien-3-oic acid [(+)-(4®2)-*abeo*-kolavelool-3-oic acid] (**39**)	*A. chamissonis* [41]
(5*R*,8*R*,9*S*,10*R*)-*ent*-Clerod-14-en-3*β*,4*α*,13*α*-triol [(–)-3*α*,4*β*-dihydroxykolavelool] (**40**)	*A. chamissonis* [41]
(5*R*,8*R*,9*S*,10*R*)-*ent*-Clerod-3,14-dien-13*α*-ol [(–)-kolavelool] (**41**)	*A. chamissonis* [41]; *A. cymbifera* [38]; *A. galeata* [38]
(5*R*,8*R*,9*S*,10*R*)-*ent*-Clerod-3,14-dien-2*α*,13*α*-diol [(–)-2*β*-hydroxykolavelool] (**42**)	*A. chamissonis* [41]
(5*R*,8*R*,9*S*,10*R*)-*ent*-Clerod-3,14-dien-2*β*,13*α*-diol [(+)-13-*epi*-2*α*-hydroxykolavelool; 13-*epi*-roseostachenol] (**43**)	*A. chamissonis* [41]
(5*S*,8*R*,9*S*,10*R*)-2-Oxo-*ent*-3-cleroden-15-oic acid (**44**)	*A. brasilienses* [38]
(5*R*,8*R*,9*S*,10*R*)-2-Oxo-*ent*-3-cleroden-15-oic acid [2-oxopopulifolic acid] (**45**)	*A. brasilienses* [39]; *A. cymbifera* [39]; *A. galeata* [40]
(5*R*,8*R*,9*S*,10*R*)-2-Oxo-*ent*-15-ethanoyl-3-clerodene [2-oxodihydrokolavenol acetate] (**46**)	*A. galeata* [40]
Methyl (5*R*,8*R*,9*S*,10*R*)-2-oxo-*ent*-3-cleroden-15-oate [methyl 2-oxopopulifoloate] (**47**)	*A. esperanzae* [38]
(5*R*,8*R*,9*S*,10*R*)-2-Oxo-*ent*-clerod-3,14-dien-13*α*-ol [(–)-13-*epi*-2-oxokolavelool; 13-*epi*-roseostachenone] (**48**)	*A. chamissonis* [41]
Methyl (5*S*,8*R*,9*S*,10*R*)-2-oxo-*ent*-clerod-3,13-dien-15-oate (**49**)	*A. brasilienses* [38]
(5*R*,8*R*,9*S*,10*R*)-2-Oxo-*ent*-clerod 3,13-dien-15-oic acid [Δ^13,14^-2-oxokolavenic acid] (**50**)	*A. brasilienses* [38]
Methyl (5*R*,8*R*,9*S*,10*R*)-2-oxo-*ent*-clerod-3,13-dien-15-oate [methyl Δ^13,14^-2-oxokolavenoate] (**51**)	*A. esperanzae* [38]
(5*S*,8*S*,9*R*,10*S*)-2-Oxo-*ent*-clerod-3,13-dien-15-oic acid (**52**)	*A. brasilienses* [38]
Methyl (5*R*,8*R*,9*S*,10*R*)-*ent*-2*α*-hydroperoxy-3-cleroden-15-oate (**53**)	*A. esperanzae* [38]
(5*R*,8*R*,9*S*,10*R*)-*ent*-2*α*-Hydroperoxy-clerod-3,14-dien-13*α*-ol [(–)-2*β*-hydroperoxykolavelool] (**54**)	*A. chamissonis* [41]
Methyl (5*R*,8*R*,9*S*,10*R*)-*ent*-2*α*-hydroperoxy-clerod-3,13-dien-15-oate (**55**)	*A. esperanzae* [38]

Usual names are given in brackets

## Furanoditerpene isolated from *Aristolochia* species

Analgesic and anti-inflammatory activities have been observed for furanoditerpenes [42,43], and their derivatives also show sedative [42], anticonvulsant [44], and plant growth regulatory activities [45]. Columbin (**56**) was isolated from *A. albida* [46] (Figure 6). The furan moiety is not fused to other rings, as is commonly found in several furanoditerpenes from natural products or synthesized [47]. It is the only furanoditerpene found in the genus *Aristolochia*. 

## Labdane derivatives isolated from *Aristolochia* species

Labdane diterpenoids are fungal growth regulator and plant growth inhibitor [48,49,50], showing high antibacterial activity [51]. Commercially, labdanes are used as natural fixatives, modifiers, and lotions by the perfume industry, and as a flavouring agent in the tobacco industry [52]. Table 3 shows the labdane diterpenoids isolated from *Aristolochia* species (**57** to **67** in Figure 7). Structures **58** and **59** have both been named as *ent*-labd-13-en-8*β*-ol-15-oic acid [53]. 

**Table 3:** Labdane diterpenoids isolated from *Aristolochia* species.

Clerodane	Species
(5*R*,8*R*,9*S*,10*S*)-*ent*-Labdan-8*β*-hydroxy-15-oic acid (**57**)	*A. galeata* [40]
(5*R*,8*R*,9*S*,10*S*)-*ent*-Labd-13-en-8*β*-hydroxy-15-oic acid [Δ^13,14^-*ent*-labd-8*β*-ol-15-oic acid] (**58**)	*A. galeata* [40]
(5*R*,8*R*,9*S*,10*S*)-*ent*-Labd-14-en-8*β*-ol (**60**)	*A. cymbifera* [40]
(5*S*,8*R*,9*R*,10*R*)-*ent*-Labd-14-en-8*β*,13*α*-diol (**61**)	not isolated from *Aristolochia* species [54]
(5*R*,9*S*,10*S*)-*ent*-Labd-8(17)-en-15-oic acid (**62**)	*A. ringens* [55]
(5*R*,9*S*,10*S*)-*ent*-Labd-8(17),13-dien-15-oic acid [copalic acid] (**63**)	*A. esperanzae* [40]; *A. galeata* [40]
(5*R*,9*S*,10*S*)-*ent*-Labd-6*β*-hydroxy-8(17),13-dien-15-oic acid (**64**)	*A. esperanzae* [40]
Methyl (5*R*,8*R*,9*S*,10*S*)-*ent*-labd-8(17),13-dien-15-oate [methyl copalate] (**65**)	*A. esperanzae* [40]
Methyl (5*R*,9*S*,10*S*)-*ent*-labd-6*β*-hydroxy-8(17),13-dien-15-oate (**66**)	*A. esperanzae* [40]
(5*R*,10*S*)-*ent*-Labd-8,14-diene (**67**)	*A. cymbifera* [40]

Usual names are given in brackets

## ^13^C-NMR data of diterpenes

Table 4 and Table 5 show the ^13^C-NMR data of the diterpenoids **1** to **67**. In Table 4 the ^13^C-NMR data of **17** (in CDCl_3_) were reassigned and a new structure **22** was proposed according to ^13^C-NMR data in CDCl_3_, C_5_D_5_N, and DMSO-*d*_6 _[34] (see Figure 2). The carbon chemical shifts of the kaurane diterpenoids **1** to **23** are characteristic only at region between *δ*_C_ 38.0 and 42.0 assigned to C-1 and C-10. The other carbon chemical shifts do not show any characteristic features for this skeleton type. In diterpenes **15** and **16** containing a double bond between C-15 and C-16, the carbon chemical shifts are registered near *δ*_C_ 135.0 and 145.0, respectively. On the other hand, the double bond is located between C-16 and C-17 of **17** to **23** and their carbon chemical shifts are registered near *δ*_C_ 156.0 and 103.0, respectively.

**Table 4:** ^13^C-NMR data (in CDCl_3_) of diterpenes from *Aristolochia* species.

Carbon	Compound / *δ*_C_ (in ppm)														
	**1 **[56]	**2 **[13]	**3 **[31]	**4 **[13]	**6 **[28]	**8 **[57,58]	**10 **[59]	**11 **[28]	**12 **[13]	**13 **[28]	**14 **[56]	**15 **[28]	**16 **[13]	**17 **[33]	**18 **[33]
1	42.0	39.2	40.3	40.9	40.35	41.1	40.4	39.82	40.4	40.66	41.0	40.73	42.0	39.10	41.13
2	18.6	18.2	18.6	18.6	17.97^a^	19.8	18.6	18.39^a^	18.7	19.06	18.5	19.05^a^	18.6	20.07	19.52
3	42.0	42.0	41.9	42.0	35.55	38.7	42.0	34.33	42.1	38.39	41.9	38.03	43.8	35.00	38.23
4	33.2	33.1	33.2	33.6	38.50	43.9	33.2	48.47	33.3	43.66	33.2	43.55	33.2	43.60	44.66
5	56.2	56.1^a^	56.2	56.1^a^	56.90^b^	57.0	56.2	56.68^b^	55.9	56.96	56.1	56.65	55.8	49.80	57.49
6	20.4	20.6	20.4	20.7	20.49	23.0	20.2	20.10	19.3	22.00	19.2	20.70	19.2	19.51	22.28
7	40.3	40.3	42.0	40.4	42.31	42.8	41.1	42.02	32.5	41.52	40.3	39.24	39.2	40.84	41.71
8	45.3	45.3	44.7	45.1	45.17	45.0	45.4	45.20	43.4	44.51	42.5	48.81	48.8	47.63	44.17
9	56.8	56.0^a^	56.7	56.0^a^	56.70^b^	56.3	55.9	55.20^b^	50.8	55.40	55.0	47.57	48.3	47.89	55.55
10	39.3	38.0	39.4	39.2	39.15	40.1	39.3	39.44	39.2	39.64	39.4	39.72	39.4	40.00	40.09
11	18.0	18.5	18.3	18.3	17.93^a^	19.0	19.3	18.13^a^	18.2	19.06	18.5	18.75^a^	18.6	77.52	18.43
12	26.9	31.2	26.3	31.3	26.06	26.8	29.2	26.90	27.0	27.06	29.7	25.31	25.6	33.97	33.53
13	49.0	44.7	45.5	41.4	48.64	45.9	42.7	48.93	36.0	45.64	47.9	40.92	41.1	44.41	44.28
14	37.7	40.8	37.3	38.1	37.36	37.8	38.6	37.81	36.0	37.88	37.5	43.71	40.4	39.79	40.13
15	58.0	45.0	53.4	44.7	57.66	53.9	48.9	57.80	65.7	55.54	55.2	135.39	135.7	47.41	49.39
16	79.4	55.9	81.9	45.9	79.16	81.7	66.4	79.29	69.5	89.16	222.5	145.82	145.6	155.24	156.32
17	24.5	182.5	66.4	14.8	24.05	66.5	50.4	24.47^c^	59.9	70.05	-	60.63	61.1	104.09	103.69
18	33.5	33.5	33.5	33.2	26.89	29.3	33.6	24.26^c^	33.6	28.93	33.6	28.87	35.5	30.08	29.38
19	21.6	21.5	21.5	21.6	64.98	180.1	21.6	205.80	21.6	182.64	21.7	180.90	21.5	184.05	184.00
20	18.0	17.3	17.8	17.4	18.11	16.0	17.8	16.40	17.5	15.74	18.0	15.25	17.6	15.93	16.02
1’										108.39					
2’										26.81					
3’										26.91					
Carbon	Compound / *δ*_C_ (in ppm)														
	**19 **[56]	**20** [57]^(?)^	**22 **[34]	**22 **[34] ^(P**)**^	**22** [34]^(D)^	**23 **[60]	**27 **[26]	**27 **[34]	**29 **[61]	**30 **[38]	**30 **[38]	**31 **[39]	**32 **[39]	**33 **[38]	**33 **[39]
1	41.3	40.5	39.79	40.9	40.3	38.7	18.3	17.4	17.3	17.5	18.2	17.7	17.6	17.3	18.3
2	18.7	18.3	20.07	19.7	19.0	27.6	26.8	27.6	27.5	27.1	26.8	24.1	24.0	27.5	26.9
3	42.0	35.7	40.84	38.5	37.9	80.6	120.5	120.6	120.4	120.0	120.4	123.2	123.1	120.5	120.4
4	33.3	39.3	43.60	43.7	42.5	42.7	144.5	144.4	144.4	143.7	144.4	139.9	139.9	144.5	144.4
5	56.1	56.9	49.80	49.5	46.3	55.8	38.2	38.3^a^	38.3	38.0^a^	38.4	38.5	38.2	38.3^a^	38.2
6	20.3	20.5	35.00	30.4	29.3	20.1	36.9	36.5	35.9	36.4	36.8	37.8	37.7	36.4^b^	36.8
7	40.4	41.7	77.52	76.2	75.0	41.3	27.6	27.0	26.8	26.4	27.5	28.8	28.7	26.9	27.5
8	44.2	44.0	47.63	48.9	48.0	43.9	36.2	36.3	36.1	36.0	36.1	37.3	37.2	36.4^b^	36.3
9	56.1	56.2	47.89	47.4	48.6	55.8	40.0	38.7^a^	38.1	38.3^a^	39.9	39.9	39.9	38.4^a^	38.3
10	39.3	38.7	40.00	39.5	38.8	39.6	46.4	46.6	46.4	46.1	46.3	44.5	44.5	46.6	46.5
11	18.1	18.2	19.51	18.5	17.7	18.3	35.5	35.1^b^	35.5	35.0^b^	35.4	35.1	35.0	35.0	36.3
12	33.3	33.2	33.97	34.0	33.3	33.0	29.5	35.6^b^	35.4	35.8^b^	29.4	29.4	29.3	36.9^a^	35.0
13	44.2	44.2	44.41	44.3	43.4	43.9	30.9	31.0	30.6	30.6	31.0	30.9	31.1	164.4	164.6
14	39.9	39.7	39.10	39.1	38.4	38.5	41.6	41.7	36.5	41.0	41.5	41.6	41.5	114.9	114.8
15	49.2	49.1	47.41	46.6	45.6	48.8	179.4	179.8	62.8	172.8	173.8	179.4	173.8	172.0	172.1
16	156.0	155.9	155.24	156.2	155.4	155.4	19.9	19.9	19.6	19.5	19.9	19.8	19.8	19.5	19.5
17	102.8	103.0	104.09	103.6	103.2	103.1	16.0	16.1	15.7	15.5	15.9	16.0	15.9	15.9	16.0
18	33.7	27.1	30.08	28.6	28.5	22.8	19.9	18.4	18.3	18.0	19.9	33.0	33.0	18.3	20.0
19	21.7	65.6	184.05	178.2	179.0	64.3	18.0	20.0	19.3	19.5	18.0	20.1	19.9	20.0	18.0
20	17.6	18.1	15.93	15.7	15.4	18.3	18.5	18.1	17.8	17.8	18.4	17.4	17.3	17.9	18.3
C=O									178.6						
MeCO									20.8						
OMe										50.6	51.3		51.3		
Carbon	Compound / *δ*_C_ (in ppm)														
	**34 **[62]	**35 **[63]	**36 **[38]	**37 **[38]	**38 **[40]	**39 **[41]	**40 **[41]	**41 **[41]	**42 **[41]	**43 **[41]	**44 **[38]	**45 **[40]	**46 **[40]	**47 **[38]	**48 **[41]
1	18.37	18.40	17.5	18.6	18.1	29.4	16.2	18.2	27.3^a^	28.9	35.1^a^	35.6	35.6	35.6^a^	34.3^a^
2	26.98	26.92	27.1	24.1	27.7	125.6	30.3	27.4	65.6	69.5	199.1	201.2	200.2	200.0	200.5
3	120.52	120.46	120.0	123.3	120.3	171.0	76.2	120.4	122.1	124.4	128.5	125.5	125.5	125.5	127.4
4	144.60	144.50	143.7	139.8	144.4	168.7	76.5	144.5	150.1	147.9	168.6	173.4	171.0	172.6	172.6
5	38.28	38.30	38.0^a^	37.9	38.3	50.6	38.3	38.1	38.0	38.0	38.6^c^	40.0	38.8	39.9^b^	39.7
6	36.94	36.99	36.4	37.6^a^	36.1	34.4	32.4	36.8	36.4	36.5	36.8^b^	36.0	35.5	34.8^a^	35.5
7	27.61	27.63	26.4	28.9	26.8	28.3	26.4	26.8	27.2^a^	27.2	29.0	27.0	26.9	26.9	26.8
8	36.36	36.41	36.0	37.5	36.8	37.1	36.0	36.1	36.3	35.9	37.3	36.1	36.1	36.1	35.8
9	38.72	38.72	38.3^a^	38.9	38.1	37.5	41.2	38.3	38.9	38.6	39.3^c^	38.6	38.6	38.8^b^	38.3
10	46.53	46.60	46.0	44.9	46.3	53.9	40.7	46.3	40.4	45.2	45.7	45.7	45.7	45.6	45.5
11	36.63	36.72	34.2	35.0	31.8	33.5	32.2	31.8	31.0	31.8	35.4^a^	34.9	34.9	34.9^a^	31.1
12	32.95	32.97	37.7	37.0^a^	35.2	35.5	35.4	35.3	36.4	35.2	36.2^b^	36.0	34.7	35.9^a^	34.7^a^
13	140.93	143.22	160.8	164.5	73.3	73.0	73.5	73.4	73.2	73.3	30.7	30.8	30.4	30.9	73.0
14	123.13	118.03	114.5	115.1	145.2	144.9	145.0	145.1	146.4	145.0	41.4	41.5	36.9	41.3	144.8
15	59.51	61.44	166.5	172.4	111.6	111.9	111.6	111.8	110.9	111.9	178.7	178.9	63.0	173.4	111.9
16	16.52	16.70	18.5	19.6	27.4	27.8	27.4	27.7	26.3	27.7	19.9	19.9	19.9	19.8	27.7
17	16.07	15.98	15.5	16.1	15.8	15.0	15.9	15.9	15.8	15.9	16.0	15.7	15.8	15.7	15.6
18	18.45	17.92	18.0	20.0	18.3	11.7	21.3	18.0	17.9	17.7	20.5	18.4	18.5	18.4	18.9^b^
19	20.03	19.99	19.5	33.2	19.8	16.9	17.2	19.8	18.3	19.9	32.1	18.9	18.7	18.9	18.2^b^
20	18.07	18.34	17.8	17.9	17.8	18.2	18.5	18.4	18.3	18.5	18.0	17.9	18.0	18.0	17.9
C=O		170.94											178.9		
MeCO		20.94											20.8		
OMe			50.1											51.4	
	Compound / *δ*_C_ (in ppm)														
Carbon	**49 **[38]	**50 **[38]	**51 **[38]	**54 **[41]	**56 **[46]	**57 **[40]	**58 **[64]	**60 **[40]	**61** [54]^A^	**62 **[55]	**63 **[65]	**65** (?) [66]	**66 **[40]	**67 **[40]	
1	35.4^a^	35.4^a^	35.6^a^	22.0	74.18	39.1	39.8	39.0	40.4	33.1	39.1	19.45	43.9	36.9	
2	200.3	200.0	200.2	79.2	128.68	18.2	-	18.2	19.0	21.7	19.4	22.36	19.5	19.0	
3	128.5	125.3	125.4	116.7	136.84	42.1	41.9	41.9	42.7	35.4	42.1	24.51	42.0	41.2	
4	167.5	172.3	172.7	155.0	80.48	33.1	-	33.3	33.7	39.1	33.6	33.64	34.5	33.2	
5	38.6^b^	39.8^b^	39.9^b^	37.8	37.16	55.9	56.1	55.8	56.9	36.6	55.5	55.59	57.5	51.8	
6	36.7^a^	34.8^a^	34.9^a^	33.2	25.59	18.1	23.5	18.1	21.1	28.6	24.5	32.75	69.4	19.0	
7	28.9	26.7	26.9	27.1	17.33	41.3	44.7	40.5	45.1	37.4	38.3	38.41	47.7	33.5	
8	36.6	36.1	36.1	36.4	47.58	73.3	74.4	73.3	73.9	160.6	148.3	148.17	144.0	125.4	
9	39.9^b^	38.7	38.7^b^	39.1	35.28	59.3	61.3	58.2	62.3	48.6	56.2	57.20	56.7	140.4	
10	45.7	45.6	45.8	40.4	44.49	38.9	39.2	38.8	39.8	40.0	39.7	39.80	40.9	37.2	
11	34.0	34.2^a^	34.0^a^	30.7	41.90	22.3	20.5	22.4	20.0	27.5	21.5	38.96	21.6	25.3	
12	36.8^a^	35.9^a^	35.9^a^	36.1	70.66	42.1	44.5	41.9	46.2	29.8	40.1	42.21	39.5	41.7	
13	160.3	162.6	160.3	73.9	124.79	30.9	163.9	30.9	73.3	30.8	164.3	160.69	160.7	31.1	
14	115.2	115.1	115.3	146.3	108.40	40.5	114.7	144.7	147.5	41.4	114.6	115.73	115.2	145.9	
15	167.0	171.2	167.1	111.2	139.66	177.9	171.5	111.8	110.7	179.8	171.8	166.40	167.3	112.0	
16	19.1	19.3	19.1	25.0	143.96	19.5	19.4	19.5	27.8	19.8	19.2	14.51	19.0	20.0	
17	15.9	15.5	15.7	15.6	175.48	30.5	24.0	30.4	24.5	102.5	106.4	21.73	110.3	19.4	
18	20.5	18.2	18.4	18.3	172.37	21.5	21.5	21.5	33.7	18.2	33.6	25.26	23.6	21.5	
19	32.1	18.7	18.9	18.1	27.00	33.3	33.4	33.1	21.8	20.8	21.7	33.64	33.7	33.2	
20	17.8	17.6	17.9	18.4	24.31	-	15.4	-	15.8	15.9	14.5	106.53	17.1	19.3	
1’			50.8												
2’															
3’	50.7											50.56	50.8		

a, b, and c may be interchanged for the same structure; (*) reassigned ^13^C-NMR data in CDCl_3_; (P) ^13^C-NMR data in C_5_D_5_N; (D) ^13^C-NMR data in DMSO-d_6_; (A) ^13^C-NMR data in acetone-d_6_; (?) solvent not given

**Table 5:** ^13^C-NMR data (in CDCl_3_) of substituted diterpenes isolated from *Aristolochia* species.

Carbon	Compound / *δ*_C_ (in ppm)			Carbon	Compound / *δ*_C_ (in ppm)		
	24 [ 31]	25 [ 31]	26 [ 31]		24	25	26
1	40.3	40.4	40.4	1’	-	-	-
2	18.6	-	-	2’	112.7	112.8	112.5
3	41.9	42.0	42.0	3’	143.1	-	-
4	33.3	-	-	4’	147.5	146.8	146.2
5	56.0	56.1	56.2	4a’	-	-	-
6	20.5	-	20.5	4b’	131.0	-	131.0
7	42.1	42.0	42.0	5’	119.2	127.4	119.2
8	44.9	-	-	6’	131.0	130.5	-
9	56.7	56.6	56.5	7’	108.0	-	108.0
10	39.4	-	-	8’	156.9	130.2	156.9
11	18.3	-	-	8a’	120.2	128.5	120.2
12	26.3	27.1	27.4	9’	121.2	126.5	121.1
13	46.3	46.5	43.5	10’	145.9	-	-
14	37.2	37.5	38.1	10a’	-	118.3	119.2
15	53.3	53.4	51.0	11’	167.2	167.5	167.2
16	80.1	80.0	-	OCH_2_O	102.4	103.0	102.4
17	69.6	70.2	63.7	OMe	56.2		56.2
18	33.6	33.6	33.6				
19	21.5	22.0	21.6				
20	17.8	18.0	17.8				

(-) Data not observed

The characteristic carbon chemical shifts for clerodane derivatives (except for **39** and **40**) are observed around *δ*_C_ 120.0-123.0 and 139.0-145.0, which are assigned to C-3 and C-4, respectively, as shown in Table 4 (see Figure 4 and Figure 5). However, the carbon chemical shift ranges show higher values when C-2 is oxygened, as is the case of **42** to **55** (Figure 5). The carbon chemical shifts around *δ*_C_ 38.0-40.0 (assigned to C-5 and C-9) and *δ*_C_ 36.0-38.0 (assigned to C-6 and C-8) are registered in the ^13^C- NMR spectra of these compounds. Values close to *δ*_C_ 145.0 and 112.0 are assigned to the double bond between C-14 and C-15 in clerodane diterpenoids, as shown in **38**-**43**, **48**, and **54**. Besides, the values close to *δ*_C_ 160.0 and 115.0 were assigned to double bond between C-13 and C-14, respectively, and *δ*_C_ 73.0 for hydroxylated C-13 of these compounds. 

Structure of furanoditerpenes (Figure 6) can be confirmed by carbon chemical shifts at *δ*_C_ 124.79, 108.40, 139.66, and 143.96 assigned to C-13, C-14, C-15, and C-16, respectively for **56**. 

Despite the fact that **61** was not isolated from an *Aristolochia* species, its stereochemistry is close to that of **59** (see Figure 7). Thus, ^13^C-NMR data of **61** were included in Table 4 to provide insights about the corresponding data of **59**. The carbon chemical shifts close to *δ*_C_ 145.0 and 112.0 are assigned to double bond between C-14 and C-15 in labdanes, as shown in Table 4 for **60**, **61**, and **67**. Values close to *δ*_C_ 160.0 and 115.0 can be assigned to double bond between C-13 and C-14 in **58** and **63**-**66**.

Table 5 shows the ^13^C-NMR data of the substituted kaurane diterpenoids **24** to **26** (see Figure 3). These compounds present an aristolochic acid derivative bound to a kaurane diterpenoids at O-16 (for **24** and **25**) and O-17 (for **26**). Some carbon chemical shifts were not observed in the ^13^C NMR data of **25** and **26**. As it would be expected only the carbon chemical shifts of C-13 to C-17 of **26** are different when comparing to **24** and **25**.
